# Normal range of myocardial layer-specific strain using two-dimensional speckle tracking echocardiography

**DOI:** 10.1371/journal.pone.0180584

**Published:** 2017-06-29

**Authors:** Yasufumi Nagata, Victor Chien-Chia Wu, Yutaka Otsuji, Masaaki Takeuchi

**Affiliations:** 1 Second Department of Internal Medicine, University of Occupational and Environmental Health, School of Medicine, Kitakyushu, Japan; 2 Department of Cardiology, Chang Gung Memorial Hospital, Chang Gung University College of Medicine, Taipei, Taiwan; 3 Department of Laboratory and Transfusion Medicine, University of Occupational and Environmental Health, School of Medicine, Kitakyushu, Japan; Azienda Ospedaliera Universitaria Federico II, ITALY

## Abstract

**Background:**

Newer 2D strain software has a potential to assess layer-specific strain. However, normal reference values for layer-specific strain have not been established. We aimed to establish the normal ranges of layer-specific longitudinal and circumferential strain (endocardial global longitudinal strain (GLS), transmural GLS, epicardial GLS, endocardial global circumferential strain (GCS), transmural GCS, and epicardial GCS).

**Methods and results:**

We retrospectively analyzed longitudinal and circumferential strain parameters in 235 healthy subjects, with use of layer-specific 2D speckle tracking software (GE). The endocardial strain/epicardial strain (Endo/Epi) ratio was also measured to assess the strain gradient across the myocardium. The endocardial, transmural, and epicardial GLS values and the Endo/Epi ratio in the normal subjects were -23.1±2.3, -20.0±2.0, -17.6±1.9, and 1.31±0.07, respectively. The corresponding values of GCS were -28.5±3.0, -20.8±2.3, -15.3±2.0, and 1.88±0.17, respectively. The layer-specific global strain parameters exhibited no age dependency but did exhibit gender dependency except for endocardial GCS. A subgroup analysis revealed that basal and middle levels of endocardial LS was decreased in the middle and elderly aged group. However, apical endocardial LS was preserved even in the elderly subjects.

**Conclusions:**

We proposed normal reference values for layer-specific strain based on both age and gender. This detailed strain analysis provides layer-oriented information with the potential to characterize abnormal findings in various cardiovascular diseases.

## Introduction

Two-dimensional (2D) speckle tracking echocardiography (STE) derived strain imaging in an emerging method to characterize left ventricular (LV) function in health and disease [1, 2]. Deformation imaging by STE has been shown to have superior prognostic value to conventional measures for predicting major adverse cardiac event [3]. Although LV ejection fraction (LVEF) is the most often used parameter to evaluate LV mechanics, and is closely coupled to adverse cardiovascular outcomes [4], it is not sufficiently sensitive to detect subtle myocardial dysfunction [5]. Strain by 2DSTE has gained popularity, because it allows clinicians to perform a more sophisticated assessment of LV systolic and diastolic function [1, 2, 6]. Among the various strain parameters derived from 2DSTE, global longitudinal strain (GLS) is most frequently used because of its robustness and reliability to detect latent systolic dysfunction and distinguish between high-risk patients with poor prognoses and patients with benign prognoses [7–9]. The usefulness of global circumferential strain (GCS) also reported in several studies [10, 11]. The LV myocardium has a complex architecture and consists of circumferential fibers in the midwall layer and longitudinal fibers in the endocardial and the epicardial layers [1, 12]. As acquired myocardial disease processes often develop firstly in the endocardium and endocardial fibers [13], the endocardial longitudinal strain (LS) may be more sensitive than transmural LS in the detection of subtle abnormalities observed during the early stages of heart disease. Recent advancements in 2D strain software have provided the capability to measure layer-specific strain (e.g., endocardial strain or epicardial strain), the usefulness of which has been described in recent publications [14–17]. However, normal ranges for each type of layer-specific strain and the normal strain gradient from the endocardium to the epicardium have not been determined [15, 18].

Establishment of the range of reference values and associated variations of 2DSTE-derived layer-specific LV strain is a prerequisite for its routine clinical adoption. Accordingly, the aim of this study was to establish the normal ranges of LV endocardial, epicardial, and transmural GLS and GCS and the strain gradient between the endocardium and epicardium in healthy subjects.

## Methods

### Study subjects

We enrolled 254 healthy subjects (122 men, mean age: 44 years; range: 20 to 76 years) who were primarily hospital employees, relatives, and other volunteers recruited via advertising. Some of the data collected were used for analysis in the JUSTICE study [19]. The inclusion criteria were as follows: (1) age ≥20 years, (2) no history of hypertension and a normal blood pressure at the time of examination, (3) no history of diabetes mellitus, hyperlipidemia, cardiovascular disease or chronic kidney disease (defined as an estimated glomerular filtration rate <60 ml/min/1.73 m^2^) and (4) no history of cardiac medication use. All subjects underwent a physical examination and 2D transthoracic echocardiography to exclude patients with either valvular heart disease or regional wall motion abnormalities. To evaluate age and gender dependency of several strain parameters, we performed subgroup analysis according to age and gender. Specifically, we divided subjects into 5 groups according to 3^rd^, 4^th^, 5^th^, 6th and more than 7^th^ decade of life. The study was approved by the ethics committee in the University of Occupational and Environmental Health. As this was a retrospective study, the Institutional Review Board waved the requirement for informed consent.

### 2D echocardiography

Comprehensive 2D and Doppler echocardiography were performed using a commercially available ultrasound machine (Vivid 7 or Vivid E9, GE Healthcare, Horten, Norway). Three levels (basal, middle and apical) of LV short axis views and three LV apical views (4-chamber, 2-chamber and long-axis views) were acquired in the left lateral decubitus position during a breath hold. Both the gain and compression were adjusted to minimize the dropout of the LV endocardial and epicardial borders. The depth and sector angle were adjusted to include the entire LV; however, the sector size was minimized to maintain a higher frame rate. Using B-mode images, LV end-diastolic and end-systolic dimensions, interventricular septal thickness, and posterior wall thickness were each measured. LV mass was calculated using the formula proposed by Devereux et al. and corrected by body surface area (BSA) to derive the LV mass index [20]. LV end-diastolic and end-systolic volumes and LVEF were calculated using biplane disk-summation algorithm [21], and the indexed by BSA. Pulse-wave Doppler examination of LV inflow and outflow and tissue Doppler examination at the mitral annulus was performed according to the ASE recommendations[22]. Datasets were digitally stored on a hard disk for offline analysis.

### 2D speckle tracking echocardiography

**Fig 1:**
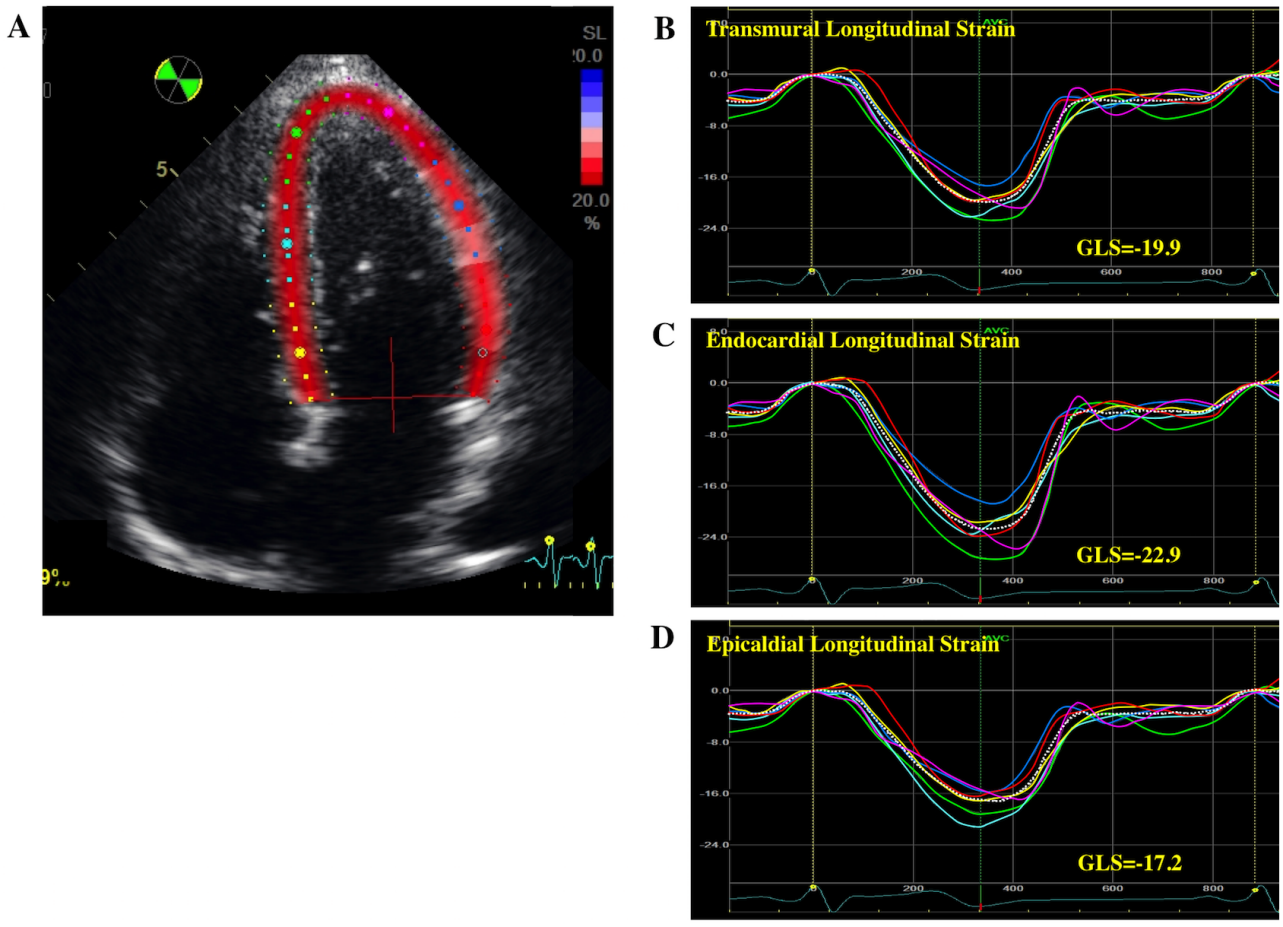
Representative layer-specific speckle tracking analysis of longitudinal strain. A: an apical 4 chamber view with color coded regions of interest, B: transmural longitudinal strain (LS) curve, C: endocardial LS curves, D: epicardial LS curves.

**Fig 2:**
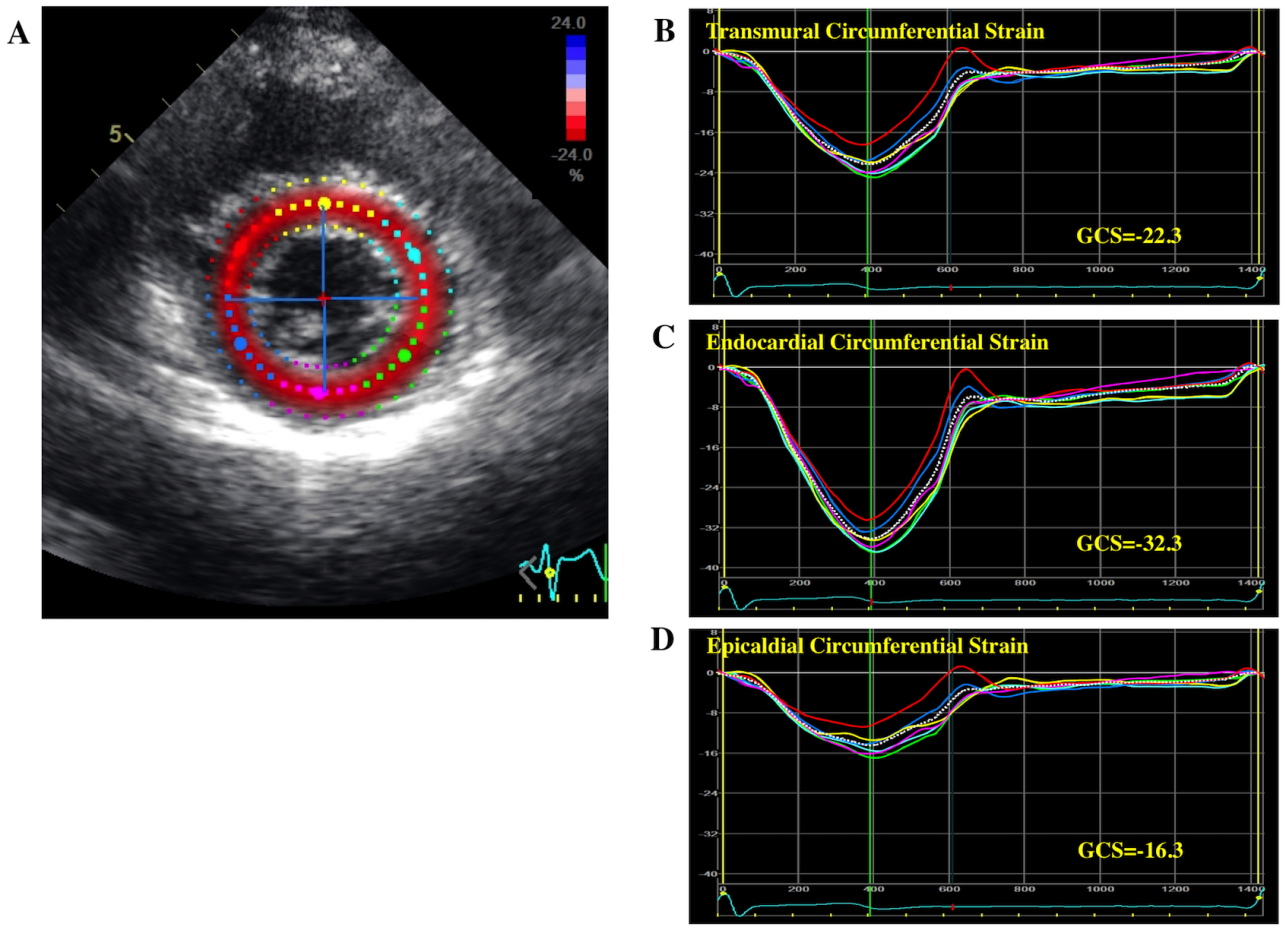
Representative layer-specific speckle tracking analysis of circumferential strain. A: a short axis view at the middle level of left ventricle with color coded regions of interest, B: transmural circumferential strain (CS) curve, C: endocardial CS curves, D: epicardial CS curves.

A 2D speckle tracking analysis was retrospectively performed using vendor-specific 2D speckle tracking software (EchoPAC PC, version 113.0.5, GE Healthcare, Horten, Norway) by an experienced observer blinded to the subject’s information. Manual tracings of the endocardial border during end-systole in three apical views and three levels of the short axis views were performed to measure longitudinal and circumferential strain. The region of interest (ROI) was then adjusted to encompass the entire thickness of the myocardium. This advanced software has a capability to adjust the regional width of ROI according the heterogeneity of myocardial thickness. The software performed a speckle tracking analysis on the LV myocardium on a frame-by-frame basis during one cardiac cycle, and divided the LV wall into 6 segments in each view. Finally, the software automatically generated time-domain strain curves in 6 segments with which end-systolic strain was subsequently calculated. GL(C)S was defined as the average longitudinal (circumferential) strain at end-systole in 18 segments. The adequacy of the tracking was verified visually, and if tracking was deemed suboptimal, a manual adjustment of both the endocardial and epicardial border was performed. If tracking was still judged unsatisfactory, the subjects were excluded from the analysis. In addition to an analysis of transmural LV strain, a layer-specific strain analysis was performed [23]. The software performed speckle tracking in the entire myocardium covered by the ROI. The transmural variation of the longitudinal and circumferential strain across the myocardial wall was calculated under the assumption of linear distribution. The endocardial and epicardial strains were calculated exactly on the endocardial and epicardial ROI borderlines, respectively (Figs 1 and 2). Subsequently, the values of transmural (not midwall), endocardial, and epicardial strain were obtained. The ratio of endocardial GL(C)S to epicardial GL(C)S was calculated using the endocardial GL(C)S/Epicardial GL(C)S (Endo/Epi) ratio for the assessment of the strain gradient. Relationship between layer-specific strain parameters and fundamental anthropometric variables were assessed.

### Intra- and inter-observer variability

Intra- and inter-observer variability in the measurement of global strain of transmural myocardium, endocardium, and epicardium and Endo/Epi ratio in both longitudinal and circumferential directions were assessed in 15 randomly selected subjects. To reduce recall bias, assessment of intra-observer variability was performed in random order more than 2 weeks apart. Parameters of observer variability were reported as bias, limits of agreement, %variability, intra-class correlation coefficient (ICC). %variability was defined as the absolute difference in the percentage of the mean of two measurements.

### Statistical analysis

All statistical analyses were performed using commercially available software (JMP version 11.0, SAS Institute Inc., Cary, NC, USA). Continuous variables were expressed either as means ± standard deviations or as medians (interquartile ranges) according to their data distributions. Differences in measurements between two groups were assessed using Student’s t test for continuous variables and either the chi-square test or Fisher’s exact test for categorical variables where appropriate. One-way analysis of variance with a post-hoc Turkey analysis was performed to compare values across more than three groups. Pearson’s correlation coefficient, r, was calculated to assess the relationship between two continuous variables. P values <0.05 were considered statistically significant.

## Results

### Study populations

Nineteen healthy subjects were excluded due to poor 2D speckle tracking, yielding a study population of 235 healthy subjects. The mean frame rates on the apical view were 63±12/sec. Feasibility of speckle tracking analysis was 97% for the assessment of transmural LS, 97% for endocardial LS, 97% for epicardial LS, 97% for transmural CS, 98% for endocardial CS and 97% for epicardial CS, respectively.

### Layer-specific longitudinal strain

The demographic and fundamental echocardiographic characteristics of the healthy subjects are summarized in Table 1. The layer-specific GLS and average LS at the basal, middle, and apical levels of the LV among the 5 groups classified according to age decade are provided in Table 2.

**Table 1:** Demographics and fundamental echocardiographic characteristics of the study population across age.

Variable	Age group (years)						
	All decades n = 235	3th (20–29) n = 44	4th (30–39) n = 50	5th (40–49) n = 48	6th (50–59) n = 46	7th (60-) n = 47	p
Age (years old)	45±14	25±3	34±3	45±3	55±3	64±4	<0.0001
Gender (male)	117	22	25	24	23	23	
BSA (m^2^)	1.64±0.18	1.62±0.15	1.66±0.19	1.63±0.19	1.66±0.17	1.61±0.19	0.5785
BMI (kg/m^2^)	21.8±2.8	21.0±2.3	21.7±3.1	21.6±2.2	22.7±2.9	22.1±3.3	0.0854
HR (beats/min)	64±9	63±9	65±8	62±9	65±9	63±9	0.2882
SBP (mmHg)	122±12	119±11	121±11	118±11	122±13	130±8	<0.0001
DBP (mmHg)	72±9	67±8	71±9	71±9	76±9	77±8	<0.0001
IVST (mm)	8.5±1.5	7.6±1.1	8.1±1.2	8.5±1.2	9.2±1.3	8.8±1.8	<0.0001
PWT (mm)	8.5±1.3	7.8±1.0	8.3±1.5	8.7±1.3	9.0±1.2	8.7±1.4	0.0002
LVEDD (mm)	46±4	47±4	47±4	46±4	46±4	45±4	0.0139
LVESD (mm)	29±4	31±4	30±3	30±3	29±4	28±4	0.0001
LVEDVI (ml/m^2^)	55±10	59±10	57±10	54±8	51±8	54±11	0.0028
LVESVI (ml/m^2^)	21±5	23±6	23±5	21±5	19±5	21±6	0.0060
LVEF (%)	62±5	61±6	60±5	61±5	63±6	62±4	0.0990
LVMI (g/m^2^)	81±17	75±17	78±15	82±17	89±18	81±18	0.0013
E (cm/sec)	76±15	81±14	81±15	76±15	71±13	70±15	<0.0001
A (cm/sec)	54±17	40±9	47±10	53±15	60±12	69±17	<0.0001
E/A	1.5±0.6	2.1±0.6	1.8±0.4	1.5±0.4	1.2±0.3	1.1±0.3	<0.0001
DcT (msec)	190±42	178±30	184±43	184±35	187±34	215±53	<0.0001
E’	10.5±2.8	13.2±2.1	11.4±2.3	10.5±2.0	9.2±2.7	8.1±2.2	<0.0001
E/E’ ratio	7.7±2.2	6.3±1.6	7.3±1.8	7.4±1.9	8.3±2.4	9.0±2.3	<0.0001

ANOVA analysis of variance, BSA = body surface area, BMI = body mass index, DcT = deceleration time, DBP = diastolic blood pressure, HR = heart rate, IVST = Interventricular septum thickness, LVEDD = LV end-diastolic dimension, LVEDVI = LV end-diastolic volume index, LVEF = LV ejection fraction, LVESD = LV end-systolic dimension, LVESVI = LV end-systolic volume index, LVMI = LV mass index, SBP = systolic blood pressure, PWT = posterior wall thickness.

Data are expressed as mean ± SD or number.

**Table 2:** Layer-specific longitudinal strains of the study population across age.

Variable	Age group (years)						
	All decades n = 235	3th (20–29) n = 44	4th (30–39) n = 50	5th (40–49) n = 48	6th (50–59) n = 46	7th~ (60-) n = 47	p
Global							
Transmural GLS (%)	-20.0±2.0	-20.0±1.7	-20.2±2.2	-20.4±2.3	-20.0±2.1	-19.4±1.7	0.1694
Endocardial GLS (%)	-23.1±2.3	-23.1±1.9	-22.8±2.7	-23.6±2.6	-23.3±2.3	-22.6±1.9	0.2599
Epicardial GLS (%)	-17.6±1.9	-17.5±1.5	-17.6±2.2	-18.2±2.1	-17.6±2.0	-17.1±1.6	0.1557
Endo /Epi ratio	1.31±0.07	1.32±0.06	1.30±0.06	1.30±0.07	1.33±0.08	1.32±0.07	0.2031
Base							
Transmural LS (%)	-18.3±2.2	-18.7±1.9	-18.7±2.4	-18.7±1.9	-18.0±1.9	-17.4±2.3	0.0054
Endocardial LS (%)	-19.3±2.3	-19.8±2.3	-19.7±2.3	-19.7±2.1	-18.7±2.2	-18.4±2.2	0.0035
Epicardial LS (%)	-17.3±2.1	-17.5±1.9	-17.5±2.4	-17.8±1.9	-17.2±2.0	-16.7±2.0	0.1370
Endo /Epi ratio	1.11±0.07	1.14±0.07	1.13±0.05	1.11±0.06	1.09±0.07	1.10±0.08	0.0047
Middle							
Transmural LS (%)	-19.5±1.9	-19.8±1.9	-19.8±2.0	-20.0±2.2	-19.4±1.7	-18.8±1.6	0.0207
Endocardial LS (%)	-21.4±2.1	-21.8±2.2	-21.5±2.1	-21.9±2.3	-21.2±1.8	-20.5±1.7	0.0069
Epicardial LS (%)	-18.0±1.8	-18.0±1.7	-18.2±2.0	-18.5±2.0	-17.8±1.7	-17.3±1.6	0.0258
Endo /Epi ratio	1.19±0.07	1.21±0.06	1.18±0.07	1.19±0.06	1.19±0.09	1.19±0.06	0.1920
Apex							
Transmural LS (%)	-22.2±3.5	-21.7±2.7	-21.4±3.7	-22.9±3.9	-22.8±4.0	-22.3±3.1	0.1492
Endocardial LS (%)	-28.9±4.6	-28.2±3.7	-27.5±4.9	-29.5±4.9	-30.3±4.7	-29.2±4.3	0.0286
Epicardial LS (%)	-17.5±2.9	-17.1±2.2	-17.1±3.1	-18.1±3.3	-17.8±3.4	-17.4±2.4	0.3072
Endo /Epi ratio	1.66±0.16	1.66±0.15	1.61±0.12	1.63±0.14	1.72±0.19	1.68±0.17	0.0106

ANOVA analysis of variance, Endo = endocardium, Epi = epicardium, GLS = global longitudinal strain, LS = longitudinal strain,

Data are expressed as mean ± SD

#### Global transmural, endocardial, and epicardial longitudinal strain

Regarding global longitudinal strain, endocardial GLS was the highest, followed by transmural GLS; epicardial GLS was the lowest in all subjects as expected. The Endo/Epi GLS ratio as a surrogate of the GLS gradient from the endocardium to the epicardium was 1.3, which indicated that endocardial GLS was approximately 30% higher than epicardial GLS.

#### Longitudinal strain at basal, middle, and apical left ventricle

A significant longitudinal strain gradient from the base toward the apex was observed with respect to both endocardial LS and transmural LS but not epicardial LS. Epicardial LS was similar between the base and the apex, while endocardial and transmural LS were increased from the base to the apex. Regarding the gradient between endocardial LS and epicardial LS described as Endo/Epi ratio, the value was increased from 1.11 at the base to 1.66 at the apex.

#### Effect of age on longitudinal strain

**Fig 3:**
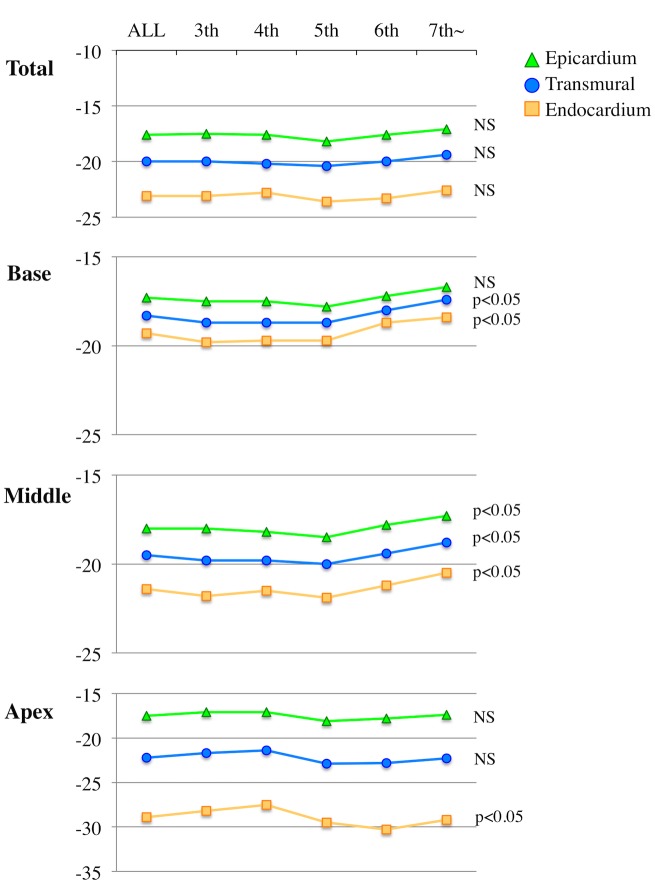
Layer-specific strain parameters in normal subjects according to age. The subjects were divided into 5 groups according to age. Layer-specific and transmural longitudinal strain are shown according to age decades in whole and 3 levels of the left ventricle.

No significant age dependency was observed with respect to all layer-specific GLS. However, both basal endocardial and transmural LS parameters, as well as all 3 parameters at the middle level, exhibited significant age dependency characterized by decrease in LS values after the 5^th^ decade of life (Fig 3). In contrast, both apical endocardial and epicardial LS parameters did not change according to the advanced aging, and the values were preserved even in the older groups (6^th^ and more than 7^th^ decades of life).

#### Effect of gender on longitudinal strain

Table 3 depicts the effects of gender on layer-specific strains. All layer-specific strain values were significantly higher in the female subjects compared with the male subjects. The Endo/Epi ratio of the female subjects was slightly but significantly lower than that of the male subjects. A linear correlation between the fundamental anthropometric and hemodynamic variables and the layer-specific GLS values is shown in Table 4. Age and heart rate did not correlate significantly with any layer-specific strain values. Gender, BSA, Body mass index and systolic blood pressure exhibited significant correlations, with r values ranging from 0.15 to 0.31.

**Table 3:** Demographics, hemodynamics parameters, and layer-specific strains of the study population across gender.

Varialble	Male n = 117	Female n = 118	P
Age	43±14	44±15	0.9495
BSA (m2)	1.76±0.13	1.51±0.13	<0.0001
BMI (kg/m2)	22.5	21.1	0.0003
HR (beats/min)	62±8	66±9	0.0007
SBP (mmHg)	125±10	119±12	0.0003
DBP (mmHg)	73±9	71±9	0.1386
LVEDVI (ml/m2)	56±10	53±9	0.0065
LVESVI (ml/m2)	22±6	20±4	0.0031
LVEF (%)	61±5	62±5	0.0696
LVMI (g/m2)	87±17	74±16	<0.0001
Longitudinal strain			
Transmural GLS (%)	-19.4±1.9	-20.6±1.9	<0.0001
Endocardial GLS (%)	-22.5±2.4	-23.7±2.1	0.0002
Epicardial GLS (%)	-17.0±1.7	-18.3±1.8	<0.0001
Endo /Epi ratio	1.33±0.08	1.29±0.05	<0.0001
Circumferential strain			
Transmural GCS (%)	-20.4±2.1	-21.2±2.1	0.0143
Endocardial GCS (%)	-28.4±3.3	-28.6±2.7	0.5088
Epicardial GCS (%)	-14.8±2.0	-15.7±2.0	0.0005
Endo /Epi ratio	1.93±0.17	1.83±0.16	<0.0001

Abbreviations were previously described

Data are expressed as mean ± SD

**Table 4:** Linear correlation between anthropometric and hemodynamic variables and layer-specific global longitudinal and circumferential strains in study subjects.

Variables	Transmural GLS		Endocardial GLS		Epicardial GLS		Endo/Epi GLS ratio	
	r	p	r	p	r	p	r	p
Age	0.08	0.2349	0.04	0.5516	0.09	0.2009	0.07	0.2669
Gender	0.27	<0.0001	0.21	0.0002	0.31	<0.0001	0.23	<0.0001
BSA	0.27	<0.0001	0.23	0.0007	0.29	<0.0001	0.16	0.0164
BMI	0.17	0.0138	0.16	0.0149	0.15	<0.0001	-0.00	0.9697
HR	0.04	0.5145	-0.04	0.8521	0.06	0.4066	0.08	0.21
SBP	0.20	0.0033	0.16	0.0148	0.23	0.0008	0.15	0.0234
DBP	0.16	0.0218	0.10	0.1331	0.18	0.0081	0.17	0.0106
Variables	Transmural GCS		Endocardial GCS		Epicardial GCS		Endo/Epi GCS ratio	
	r	p	r	p	r	p	r	p
Age	-0.09	0.2220	-0.16	0.0141	-0.04	0.5539	0.11	0.0914
Gender	0.14	0.0137	0.04	0.5065	0.20	0.0005	0.24	<0.0001
BSA	0.16	0.0228	0.05	0.4396	0.21	0.0018	0.24	0.0003
BMI	0.08	0.2635	-0.01	0.9477	0.11	0.1036	0.16	0.0215
HR	0.005	0.4675	0.00	0.9561	0.08	0.2189	0.10	0.1369
SBP	0.08	0.2436	0.00	0.9594	0.14	0.0433	0.20	0.0037
DBP	0.05	0.4675	0.00	0.6579	0.13	0.0562	0.16	0.0184

Abbreviations were previously described

### Layer-specific circumferential strain

The layer-specific GCS and average CS at the basal, middle, and apical levels of the left ventricle among the 5 groups classified according to age decade are provided in Table 5.

**Table 5:** Layer-specific circumferential strains of the study population across age.

Variable	Age group (years)						
	All decades n = 235	3th (20–29) n = 44	4th (30–39) n = 50	5th (40–49) n = 48	6th (50–59) n = 46	7th~ (60-) n = 47	p
Global							
Transmural GCS (%)	-20.8±2.3	-20.5±2.6	-21.0±2.5	-20.5±2.1	-20.8±2.1	-21.2±2.1	0.4949
Endocardial GCS (%)	-28.5±3.0	-27.9±3.2	-28.4±3.3	-28.2±2.9	-28.8±2.5	-29.3±2.9	0.1678
Epicardial GCS (%)	-15.3±2.0	-15.1±2.5	-15.5±2.1	-15.0±1.7	-15.1±1.9	-15.6±1.9	0.5368
Endo /Epi ratio	1.88±0.17	1.86±0.21	1.85±0.15	1.88±0.14	1.92±0.18	1.89±0.17	0.2962
Base							
Transmural CS (%)	-19.5±2.8	-19.4±2.7	-19.8±2.5	-19.2±2.3	-19.5±3.4	-19.6±3.0	0.8744
Endocardial CS (%)	-26.7±3.7	-26.1±3.5	-26.6±3.4	-26.2±3.2	-27.1±4.1	-27.4±4.1	0.4184
Epicardial CS (%)	-14.1±2.4	-14.3±2.5	-14.6±2.3	-13.8±2.0	-13.7±2.7	-13.9±2.5	0.3609
Endo /Epi ratio	1.92±0.24	1.85±0.26	1.85±0.21	1.92±0.23	2.01±0.21	1.98±0.25	0.0012
Middle							
Transmural CS (%)	-19.8±2.5	-19.5±2.8	-19.8±2.4	-19.4±2.0	-19.9±2.4	-20.3±2.7	0.4271
Endocardial CS (%)	-27.1±3.3	-26.6±3.4	-26.7±3.3	-26.5±3.1	-27.6±2.7	-28.1±3.7	0.0830
Epicardial CS (%)	-14.4±2.2	-14.4±2.8	-14.4±2.1	-14.0±1.7	-14.5±2.1	-14.7±2.3	0.6214
Endo /Epi ratio	1.90±0.22	1.89±0.26	1.85±0.20	1.91±0.22	1.93±0.24	1.92±0.19	0.4028
Apex							
Transmural CS (%)	-23.1±3.4	-22.5±3.7	-23.4±3.8	-22.9±3.8	-23.0±3.1	-23.8±2.6	0.4921
Endocardial CS (%)	-31.7±4.4	-30.9±4.6	-31.9±4.9	-31.6±4.5	-31.5±3.8	-32.7±4.1	0.4383
Epicardial CS (%)	-17.3±3.2	-16.8±3.5	-17.4±3.4	-17.2±3.3	-17.1±3.1	-18.2±2.8	0.2937
Endo /Epi ratio	1.86±0.22	1.88±0.23	1.86±0.21	1.86±0.20	1.87±0.25	1.82±0.23	0.6918

ANOVA analysis of variance, Endo = endocardium, Epi = epicardium, GCS = global circumferential strain, CS = circumferential strain,

Data are expressed as mean ± SD

#### Global transmural, endocardial, and epicardial strain

Similar to GLS, endocardial GCS was the highest, followed by transmural GCS and epicardial GCS was lowest in all subjects. The Endo/Epi GCS ratio was approximately 1.9, which was a higher value compared to Endo/Epi GLS ratio. In a word, endocardial GCS was approximately 90% higher than epicardial GCS.

#### Circumferential strain at basal, middle, and apical left ventricle

All layer-specific CS at apex were higher than those at base and middle, and no significant difference of layer-specific strains was observed between base and middle. Trend of strain gradient from the base toward the apex was similar in all layer-specific CS, resulting constant value of Endo/Epi CS ratio (1.9) in each layer.

#### Effect of age on circumferential strain

**Fig 4:**
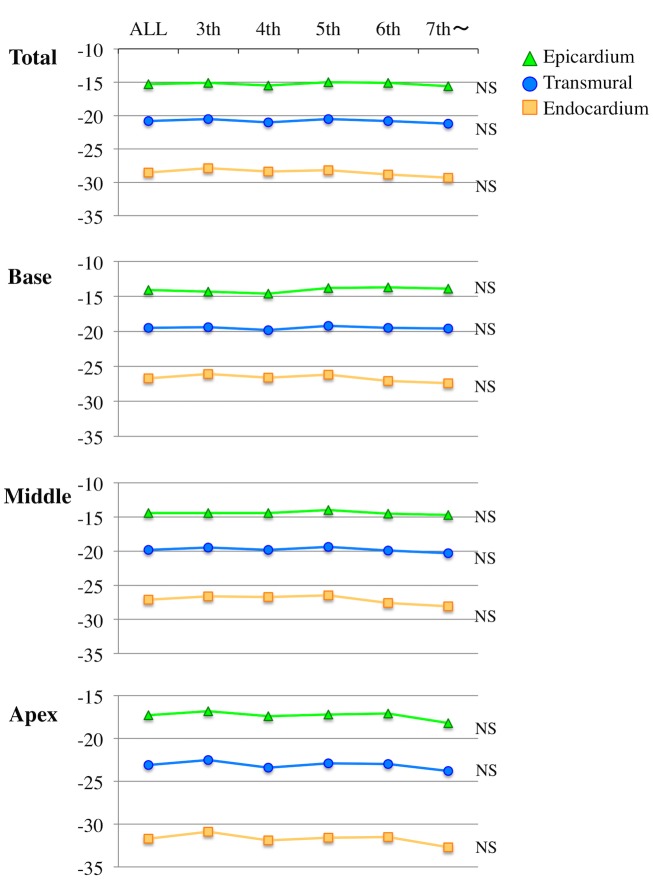
Layer-specific circumferential strain parameters in normal subjects according to age. The subjects were divided into 5 groups according to age. Layer-specific and transmural circumferential strain are shown according age decades in whole and 3 levels of the left ventricle.

Similar to GLS, there was not significant age dependency in all layer-specific GCS (Fig 4). They did not have significant differences among age decades, even when the level of LV was taken into consideration. In terms of Endo/Epi ratio, only Endo/Epi CS ratio of endocardium at the base showed significant age differences characterized by an increase in the ratio after 5^th^ decade of life.

#### Effect of gender on circumferential strain

Table 3 depicts the effects of gender on layer-specific strains. Transmural and epicardial strain values were significantly higher in the female subjects compared with the male subjects. The Endo/Epi ratio of the female subjects was significantly lower than that of the male subjects. A linear correlation between the fundamental anthropometric and hemodynamic variables and the layer-specific GCS values is shown in Table 4. Age had a weak negative correlation with endocardial GCS. Gender and BSA exhibited weak but significant correlations with all layer-specific circumferential parameters except endocardial GCS. Only Endo/Epi ratio had a relationship with both systolic and diastolic blood pressure.

### Observer variability

Intra- and inter-observer variability for layer-specific GLS and GCS was described in Table 6. Observer variabilities were lower for GLS measurements than those for GCS measurements. However, % variability of GCS was still less than 7%.

**Table 6:** Intra- and inter-observer variability in layer-specific global strain and Endo/Epi ratio.

	Global longitudinal strain			
	Bias	Limits of agreement	% variability	ICC
Intra-observer variability				
Transmural	-0.33	±1.56	2.46%	0.93
Endocardial	-0.37	±2.04	2.92%	0.89
Epicardial	-0.27	±1.45	2.47%	0.94
Endo/Epi ratio	0.002	±0.096	1.90%	0.65
Inter-observer variability				
Transmural	-0.91	±1.18	3.24%	0.89
Endocardial	-1.00	±1.33	3.09%	0.89
Epicardial	-0.97	±1.18	3.96%	0.85
Endo/Epi ratio	-0.018	±0.049	1.62%	0.77
	Global circumferential strain			
	Bias	Limits of agreement	% variability	ICC
Intra-observer variability				
Transmural	-0.79	±1.91	5.15%	0.77
Endocardial	-1.30	±3.10	4.80%	0.72
Epicardial	-0.67	±1.87	6.74%	0.77
Endo/Epi ratio	-0.002	±0.289	5.93%	0.87
Inter-observer variability				
Transmural	0.30	±2.55	5.31%	0.65
Endocardial	0.15	±4.10	5.83%	0.71
Epicardial	0.33	±2.22	6.79%	0.66
Endo/Epi ratio	0.031	±0.341	6.10%	0.80

ICC = Intra-class correlation coefficient

## Discussion

To the best of our knowledge, this was the first study to describe normal ranges for layer-specific GLS and GCS in a relatively large number of healthy subjects with approximately equal proportions of gender and age distributions. The primary findings of this study are summarized as follows: (1) gender dependency was observed for majority of layer-specific GLS and GCS values, whereas age dependency was not observed in all layer-specific GLS and GCS; (2) the Endo/Epi GLS and GCS ratio remained constant across all age groups. The Endo/Epi ratio of the female was lower than that of the male; (3) regarding the LS at each LV level, endocardial LS at the basal and the middle levels was significantly decreased after the 5^th^ decade of life, a finding reflective of aging process of endocardial function.

### Layer-specific longitudinal strain analysis

Myocardial heterogeneity is characterized by the presence of significantly higher deformation amplitude in the endocardial layer compared with the epicardial layer, which has been established using both cardiac magnetic resonance (CMR) tagging and echocardiography [15, 23, 24]. We observed a longitudinal strain gradient from the endocardial layer to the epicardial layer, resulting in an Endo/Epi strain ratio of approximately 1.3. This value was consistent with those observed in previous 2DSTE studies [15, 23]. Potential causes of the transmural strain gradient include transmural differences in wall stress, characterized by increases in end-diastolic wall stress toward the endocardium. Therefore, the endocardial fibers are stretched longer than the epicardial fibers during end-diastole, which results in increased fiber shortening in the endocardial layer during systole [25]. An additional explanation for the transmural strain gradient involves differences in coronary perfusion and metabolism between the endocardial layer and epicardial layer [26]. Experimental studies have noted higher metabolic rates, greater oxygen extraction, and greater coronary flow in the endocardium than in the epicardium [27, 28].

#### Effect of age on layer-specific longitudinal strain

We did not observe age dependency with respect to either layer-specific GLS or the Endo/Epi ratio. However, more detailed analysis demonstrated that endocardial LS decreased at both the basal and middle levels after the 5^th^ decade of life. By contrast, endocardial LS at the apical level remained constant across the all age groups. These results suggest that the basal and middle endocardium may be more prone to develop subtle changes in LV mechanics as aging process. Both functional and anatomical abnormalities, including myocardial deformation and fibrotic changes, reportedly begin in the basal LV subendocardium in some acquired heart diseases [29–31]. Furthermore, these abnormalities are closely linked to longitudinal function because endocardial fiber runs longitudinally [32, 33]. These results support the findings of our study. No age dependency of apical endocardial LS may have been due to either compensatory mechanisms or geometric changes, such as surface curvature.

#### Effect of gender on layer-specific longitudinal strain

Female gender was associated with higher strain values across the all layer-specific strains and smaller strain gradient compared with male gender in this study. The results are in agreement with previous non-layer-specific strain analyses [34]. However, the mechanism underlying these findings remains unclear [19, 34]. Many features such as lower systolic blood pressure, smaller BSA, ventricular size and LV mass of female compared to male have favorable effects on LV systolic function. Therefore, these composite factors may result in higher GLS in female than those in male. Future studies investigating the neurohormonal and biological effects of gender differences on myocardial function are warranted. Although it is physiologically important to elucidate the cause of gender differences, the small differences of strain parameters between the male and the female observed in this study would have less impact on diagnosis and decision-making of treatment in clinical settings.

### Layer-specific circumferential strain analysis

Similar to layer-specific longitudinal strain, endocardial CS value was higher than epicardial CS value. The amplitude of strain in the endocardial layer was approximately 1.9 times higher than those in epicardial layer. The degree of this difference between endocardial and epicardial strain was larger in CS compared with LS. This finding is in agreement with the previous studies using CMR tagging or echocardiography [15, 18, 24, 35]. Transmural differences in wall stress or coronary perfusion could have some influence on transmural gradient in circumferential strain as with longitudinal strain. As the other possible explanation, previous CMR tagging study has suggested that decreases in circumferential radius of curvature during systole are larger in endocardium than in epicardium, resulting higher endocardial strain [24]. This change is more marked in circumferential direction compared with longitudinal direction. Therefore, larger transmural stain gradient was observed in circumferential strain compared with longitudinal strain, leading to larger Endo/Epi GCS ratio (1.88±0.17) than Endo/Epi GLS ratio (1.31±0.07) in this study. Consistency of Endo/Epi CS ratio was observed in all level of LV, which was different from longitudinal strain. This finding was similar to the previous studies and might be explained by the similar circular shape from basal short axis view to the apical one [15, 24, 36].

#### Effect of age on layer-specific circumferential strain

No age dependency was observed with respect to any layer-specific GCS. Limited number of studies was performed to investigate the influence of aging on the CS, even in the traditional full thickness or midwall strain [34, 37]. Cheng et al reported that no significant influence of age on CS was observed in a large cohort study using 2DSTE [34]. The recent study using novel CMR feature tracking analysis on 150 healthy subjects also demonstrated that no significant age dependency was observed in transmural and endocardial GCS [37]. These findings are consistent with the result in this study. While detailed analysis according to levels of left ventricle showed some significant age dependency in layer-specific LS, no significant trend was not observed in layer-specific CS at each level of the left ventricle. This finding might imply that the impact of aging process on myocardium is greater in LS, particularly endocardium, than layer-specific CS. The established concept that endocarudium is more sensitive to external loads such as pressure than epicardium [29, 31] and the architecture of LV characterized by the longitudinally running fiber in endocardium [1, 12] could support our results.

#### Effect of gender on circumferential layer-specific strain

Significantly higher transmural and epicardial GCS and lower End/Epi ratio in female subjects than those in male subjects were observed. However, endocardial GCS had no significant differences between the gender. Conflicting results have been reported regarding the effect of gender on full-thickness CS [34, 37–39]. Regarding layer-specific strain, Andre et al. reported endocardial GCS was higher in female subjects compared with male subjects in CMR feature tracking study [37], which is inconsistent with the result in our study. Although different techniques might lead to this discrepancy, the number of study was too small to draw definite conclusions. Similar to LS, the small differences of layer-specific GCS between male and female observed in this study may be meaningless in clinical settings.

## Study limitations

This study was characterized by several limitations. First, we did not validate the accuracy of the 2D layer-specific strain measurements against reference standard such as CMR in our study subjects. Second, we could not exclude the possibility that some subjects have coronary artery disease. However, it is ethically impossible to perform invasive coronary angiography or coronary computed tomography in asymptomatic healthy subjects. Third, we did not compare the value of layer-specific strain between the different ultrasound vendors. Inter-vendor variability exists even in full-thickness strain due to differences of analytical algorithm. To make layer-specific strain clinical use, inter-vendor variability of layer-specific strain analysis should be investigated. Forth, this was a retrospective analysis which may call some selection bias. Fifth, this study did not cover the whole age group of subjects, which could not support generalizability of the results. Lastly, we did not include patients with ischemic heart disease (IHD). Clinical utility of 2D layer-specific STE for detection of IHD have recently been reported in several studies [16, 18, 23]. Further studies should be required to determine the usefulness of measuring layer-specific strain in various clinical settings.

## Conclusions

We proposed normal reference values for layer-specific strain based on both age and gender. A layer-specific strain analysis provides layer-oriented information with the potential to characterize abnormal findings in the setting of cardiovascular disease.

## Supporting information

S1 File Manuscript data of layer-specific strain in 235 healthy subjects. (XLSX)
